# Associations between psychosocial wellbeing and experience of gender-based violence at community, household, and intimate-partner levels among a cross-sectional cohort of young people living with and without HIV during COVID-19 in Cape Town, South Africa

**DOI:** 10.1186/s12889-023-16945-5

**Published:** 2023-10-27

**Authors:** Miriam Hartmann, Danielle Giovenco, Zangin Zeebari, Gina Itzikowitz, Anna Mia Ekström, Anna Nielsen, Audrey Pettifor, Linda-Gail Bekker, Anna E. Kågesten

**Affiliations:** 1 Department of Global Public Health, Karolinska Institutet, Stockholm, Sweden; 2 Women’s Global Health Imperative, RTI International, Berkely, CA USA; 3 Desmond Tutu HIV Centre, University of Cape Town, Cape Town, South Africa; 4 Jönköping International Business School, Jönköping University, Jönköping, Sweden; 5 Department of Infectious Diseases, South General Hospital, Stockholm, Sweden; 6 Department of Epidemiology, University of North Carolina at Chapel Hill, Chapel Hill, USA

**Keywords:** Violence, Gender-based Violence, HIV, young people, COVID-19, COVID-19-related stress, Mental health, South Africa

## Abstract

**Background:**

Growing evidence indicates that gender-based violence (GBV) increased during COVID-19. We investigated self-reported impact of the pandemic on GBV at community, household and intimate partner (IPV) levels among young people and its associations with psychosocial wellbeing, i.e., COVID-related stressors and mental health.

**Methods:**

Cross-sectional data were drawn from a survey with young people ages 13–24 (N = 536) living with HIV (YPLWH) and without HIV (YPLWoH), in peri-urban Cape Town, South Africa. The survey, conducted February-October 2021, examined the impact of the initial lockdown on experience and perceived changes in GBV at each level, and pandemic-related psychosocial wellbeing. Descriptive statistics and binomial and multinomial regression analyses were conducted to illustrate exposure and perceived changes in GBV since lockdown, and their association with COVID-related stress factors (e.g., social isolation, anxiety about COVID), mental health (e.g., depression, anxiety), and other risk factors (e.g., age, gender, socioeconomic status) by HIV status.

**Results:**

Participants were 70% women with mean age 19 years; 40% were living with HIV. Since lockdown, YPLWoH were significantly more likely than YPLWH to perceive community violence as increasing (45% vs. 28%, p < 0.001), and to report household violence (37% vs. 23%, p = 0.006) and perceive it as increasing (56% vs. 27%, p = 0.002) (ref: decreasing violence). YPLWoH were also more likely to report IPV experience (19% vs. 15%, p = 0.41) and perception of IPV increasing (15% vs. 8%, p = 0.92). In adjusted models, COVID-related stressors and common mental health disorders were only associated with household violence. However, indicators of economic status such as living in informal housing (RRR = 2.07; 95% CI = 1.12–3.83) and food insecurity (Community violence: RRR = 1.79; 95% CI = 1.00-3.20; Household violence: RRR = 1.72; 95% CI = 1.15–2.60) emerged as significant risk factors for exposure to increased GBV particularly among YPLWoH.

**Conclusions:**

Findings suggest that for young people in this setting, GBV at community and household levels was more prevalent during COVID-19 compared to IPV, especially for YPLWoH. While we found limited associations between COVID-related stressors and GBV, the perceived increases in GBV since lockdown in a setting where GBV is endemic, and the association of household violence with mental health, is a concern for future pandemic responses and should be longitudinally assessed.

**Supplementary Information:**

The online version contains supplementary material available at 10.1186/s12889-023-16945-5.

## Introduction

Notwithstanding growing evidence indicating that gender-based violence (GBV) increased during the COVID-19 pandemic in many settings [1], South Africa has one of the world’s highest burdens of GBV in sub-Saharan Africa. This massive public health issue is highly influenced by gender and inequity [2], but also driven by factors such as poverty, poor mental health and substance abuse [3]. Defined as any violent act that is perpetrated against a person’s will and is based on gender norms and unequal power relationships, GBV takes on many forms and is inclusive of physical, sexual, emotional, financial, or structural violence and can occur at the interpersonal, household, and community levels [4]. While the gendered nature of intimate partner violence or domestic violence in the household is commonly understood as an expression of men using power over women, gender norms and power inequalities also contribute to violence against boys and young men, including within community violence. Violence at the community level is, for example, often driven by harmful norms of masculinity, which encourage men and boys to use violence as a means of demonstrating power over other men and women alike and to gain resources to fulfill their roles of masculine provider [5]. Although women and girls tend to be at higher risk for GBV in general, evidence from South Africa indicates that experience of lifetime physical GBV among adolescents may be relatively equal across sexes albeit with different perpetrators and risk settings [6]. Recognizing the relationship between violence and gender, but also other contributors to violence, we will use the terms GBV and violence interchangeably in the remainder of the manuscript.

The concept of intersectionality is also of critical importance in understanding vulnerability to GBV, both globally and in South Africa [7, 8]. Intersectionality, which was originally used to describe intersecting oppressions of race and gender for African American women by Crenshaw [9], can be used to understand intersecting vulnerabilities based on race, class, nationality, as well as critical health related identities, such as HIV status [10]. Intersecting with generalized vulnerability to GBV, young people living with HIV (YPLWH) may be at increased risk of GBV due to stigma associated with the disease or may have been infected with HIV due to past exposure to GBV [11, 12]. In a country with the largest HIV prevalence in the world (7.3 million, 18.3% of the population over 15 years of age), this increases the importance of understanding the effects of the pandemic on GBV among young people, particularly with regards to how HIV status may interact with vulnerability to violence. It is also in line with at least two frameworks proposed to study the effects of COVID on GBV [13, 14].

Finally, adding to the importance of this issue is the fact that exposure to GBV– particularly repeated exposure – may create lasting psychological trauma that impacts young people’s psychosocial wellbeing including their mental health and related ability to cope with future stress. This includes regulation of impulsive behavior, which may continue a cycle of violence [15–17]. In the face of added psychosocial stressors of COVID-19 (henceforth referred to as ‘COVID-related stressors’) – which early evidence suggests may contribute to collective trauma [18, 19] and has been blamed for the increases in GBV during the pandemic [20] – it is also critical that we understand young people’s experience of such stressors and their association with violence at different levels of society in order to ultimately address them. Given South Africa faced one of the strictest lockdowns globally, in combination with pre-existing levels of GBV, it is one setting where this may be of particular importance. The lockdown in South Africa occurred from 26 March to 1 May 2020, when the government enforced a stay-at-home order where individuals could only leave home to purchase food or seek medical care and all sales of alcohol were banned. Restrictions began to be loosened in early May when individuals were allowed to exercise within a 5 km radius of their home between 6 and 9 am [21], however restrictions were not entirely lifted until January 2022. Evidence suggests that these restrictions had a severe impact on the South African economy and individual level economic vulnerability, a key driver of GBV exposure, intersecting with other vulnerabilities [22, 23].

In response to the above research gaps, the current study aims to: (1) examine the self-reported impact of COVID-lockdown orders on experience of violence at the community, household, and intimate-partner levels (interchangeably referred to as ‘GBV’ for simplicity within the manuscript), among young people living with and without HIV in Cape Town, South Africa; and (2) to assess the associations between GBV at different levels with psychosocial wellbeing, i.e. COVID-related stressors and mental health, as well as other risk-factors. Answering these questions may help prepare for GBV prevention and response in the face of future pandemics or other community-wide traumatic events.

## Methods

### Study design and setting

Data were drawn from the baseline survey of the *Bidirectional, Upbeat communication and Differentiated, Distanced care for Young people* (BUDDY) study, a pilot randomized controlled trial conducted in two Cape Town townships within the Klipfontein/Mitchells plain district. The primary objective of the BUDDY trial was to examine the feasibility, acceptability, and preliminary efficacy of a remote service delivery model implemented among YPLWH during the COVID-19 pandemic, and to investigate the impact of the lockdown orders on individual, socio-behavioral, structural determinants over time among young people living with and without HIV, with a focus on experiences of GBV. Details of the BUDDY study have been described elsewhere [24]. We followed the STROBE (Strengthening the Reporting of Observational Studies in Epidemiology) guidelines in designing and reporting of this observational study [25].

### Participants and sampling

Briefly, young people enrolled had to be ages 13–24, residing in the Klipfontein/Mitchells plain district and not planning to move for at least six months, have regular access to a mobile phone with SMS capacity (their own or their caregiver’s), and either self-report as HIV negative (YPLWoH) or have initiated ART at an HIV treatment facility in the district and currently be in care (YPLWH). YPLWoH were recruited through community outreach teams using flyers and street-based recruitment. YPLWH were recruited on-site at a public clinic or contacted by phone using clinic records. The target sample size for the study was 600 (300 YPLWH and 300 YPLWoH), deemed sufficient to detect a 10% change in SGBV victimization assuming 30% baseline SGBV exposure with 80% power.

### Procedures

Participants were enrolled from February-October 2021. Upon enrollment, they completed an interviewer-administered baseline survey that assessed attitudes, behaviors, and outcomes related to COVID-19, exposure to and change in GBV since lockdown, and psychosocial wellbeing in the context of the COVID-19 pandemic. GBV exposure questions covered experience of violence at community, household, and intimate partner-levels, which are described in more detail below. The survey was conducted telephonically or in-person, and data was captured via the secure web-based platform Redcap (Research Electronic Data Capture) [26].

### Measures

#### Dependent variables

The primary outcome for the current analysis, self-reported exposure to GBV, was measured using several variables at the community, household, and partner levels. All violence was asked about in relation to the March 2020 lockdown.

Community violence (CV), “Change in CV,” was a categorical variable based on the question: “Since the lockdown started (March 26, 2020), has the level of violence and crime in your neighborhood increased, decreased, or stayed the same?” (0 = decreased, 1 = stayed the same, 2 = increased).

Household-level violence (HV) was measured using two variables, with the first one “Any HV” assessing whether respondents had witnessed physical, verbal/emotional, or sexual GBV experienced by other household members since the lockdown started (0 = no, 1 = yes). Participants responded to a series of three questions asking separately about witnessing physical violence (e.g., pushed, kicked, slapped), verbal or emotional violence (e.g., humiliation or threats), and sexual violence (e.g., someone being forced to have sex against their will). These three questions represent a condensed version of standard questions asked about GBV behaviors [27]. Responding “yes” to any of these items was coded as “Any HV”. Those who had witnessed any form of HV were also asked about changes in these practices since the lockdown, used to create a second categorical variable “Change in HV” (0 = decreased, 1 = stayed the same, 2 = increased). For this variable, responses were categorized according to an approach used in the existing literature to improve comparability [28]. Following this model, if a participant responded that any form of HV had increased, their overall change in HV response was categorized as ‘increased.’ If one form stayed the same and another form decreased, their response was categorized as ‘stayed the same’. Their response was indicated as ‘decreased’ only if their responses were HV ‘decreased’ for all three forms of violence.

Finally, IPV was assessed among participants who reported having an intimate partner at the time of lockdown using the WHO Violence Against Women Instrument [27], which asks separately about specific experiences of control, emotional, physical, and sexual violent behaviors. Control included a question on restricted contact with family. Emotional abuse included questions on insults or being made to feel bad, and threats to hurt you or someone you care about. Physical abuse included slapping, hitting, kicking, dragging, pushing, shoving, choking, or burning, as well as threats to use or actual use of a knife or gun. Sexual abuse included both physical force and pressure to have sexual intercourse. An additional question on reproductive coercion was included where participants were asked about partner-related pressure or forced sex without a condom or birth control in order to get pregnant. IPV behaviors were combined into sub-forms, e.g., emotional, physical, sexual, or reproductive coercion, where a response of ‘yes’ to any of the questions in the sub-form indicated experience of that form of IPV. A combined measure of all forms, e.g. “Any IPV”, was also created. Following the series of questions, participants responding “yes” to any form were also asked if IPV had decreased, stayed the same, or increased since lockdown.

#### Independent variables

Psychosocial wellbeing was conceptualized to include several variables measuring COVID-19 related stressors, and mental health.

COVID-19 stressors included measures of social isolation and anxiety about COVID-19 illness. *Social isolation* was measured using 3 variables: (1) level of self-isolation (dichotomized as all or most of the time vs. some or none of the time), (2) change in contact with trusted social support (dichotomized as less contact vs. stayed the same/more contact), and (3) life perceived as lonelier due to COVID-19 (0 = no, 1 = yes). *Anxiety about COVID-19 illness* was measured by asking about the level of concern about becoming seriously ill from COVID-19 (dichotomized as not at all/slightly concerned vs. moderately/extremely concerned).

Mental health was measured using two validated screening tools based on self-reported symptoms of depression and anxiety. *Depression* was measured using the 9-item Patient Health Questionnaire (PHQ-9) [29]. PHQ-9 scores range from 0 to 27, with scores from 5 to 9 indicating mild depression, 10–14 moderate depression, 15–19 moderate severe depression, and 20–27 severe depression [29]. Depression was dichotomized to represent the presence of any probable clinical depression as defined as a score of 5 and above (0 = no, 1 = yes). *Anxiety* was measured using the 2-item Generalized Anxiety Disorder (GAD-2), whereby scores range from 0 to 6 with scores of 3 and above indicating an anxiety disorder [30]. Anxiety was dichotomized to represent the presence (0 = no, 1 = yes) of any probable clinical anxiety defined as a score of 3 and above. Presence of either depression or anxiety were further combined into the variable *common mental disorders* where an indication of either depression or anxiety was classified as the presence of a common mental disorder (0 = no, 1 = yes).

#### Covariates

Covariates included other risk factors for violence: age, gender identity, race, language, household type (formal or informal), intimate partnership status during lockdown, cohabitation with partner, school status, employment status, and food security. School and employment status were combined into a measure of “not in education, employment or training” (NEET) [31] whereby those participants who were not in school and were not employed were classified as NEET (0 = no, 1 = yes). Food security was asked using a question about being worried about having enough food in the past month (0 = no, 1 = yes).

### Statistical analysis

Prevalence estimates were calculated for outcome (GBV), independent (psychosocial wellbeing) and covariate (other risk factors) variables. Differences in these variables by participant gender (see Additional file 1) and HIV status were investigated using t tests for continuous variables (e.g., age) and chi-square tests for most binary and categorical variables. Fisher’s exact test was used for race, language, and partner cohabitation, where assumptions for chi-square were not met due to small cells.

Associations between independent variables – COVID-related stressors and mental health – and the dependent variables of changes in GBV outcomes were explored using crude and adjusted multinomial logistic regression models. Multinomial regression was chosen as opposed to ordinal regression because while the data could theoretically be ordered, it did not meet parallel lines assumption [32]. In line with previous violence studies [33], the lowest exposure – in this case the “decreased” category – was chosen as the reference group against which all other groups were compared, with results producing relative risk ratios (RRRs) and associated 95% confidence intervals (CIs). For example, a model comparing changes in CV would assess the relative effect of the independent variables on such violence increasing vs. decreasing, or staying the same vs. decreasing, respectively.

All models were adjusted using most covariates (i.e., age, gender, NEET status, household type, baseline food security status, and partner status during lockdown) with the exception of race, language, and partner cohabitation given the limited variability among those items within the study sample ( ≥98% agreement). In addition, the association between *any* exposure to HV during lockdowns (yes, no) with psychosocial wellbeing was explored using binomial logistic regression generating adjusted odds ratios (aORs), given the binary nature of this outcome variable. Finally, the association between independent variables and IPV was not examined given the small number of participants reporting this form of GBV. All analyses were conducted on the full sample, followed by stratification based on HIV status to explore potential differences in both prevalence of violence, as well as associated factors. A similar analysis was not done by gender given limited power and earlier findings indicating more significant differences in violence exposure by HIV status, however gender was included as a covariate. Missingness was investigated and found to be low across covariates (< 2.6%). For the outcome variable “Change in CV” approximately 10% of participants responded, ‘don’t know’. We ran a sensitivity analysis where we included ‘don’t know’ in the multinomial model and compared it to a model treating those responses as missing. Given similar results between models, a complete case analysis where ‘don’t know’ was treated as missing, was conducted. This model will be presented below. STATA v 17 [34] (StataCorp 2021, College Station, TX) was used for all analyses with significance at the alpha = 0.05 level.

### Ethical considerations

The study was reviewed and approved by the University of Cape Town Human Research Ethics Committee (HREC REF:448/2020) and the Swedish Ethical Review Authority (EPN Dnr 2020–04903). All participants provided written informed consent or assent (if under 18 years) prior to enrollment. The need for parental consent for the participants less than 18 years of age was waived by the University of Cape Town Human Research Ethics Committee (HREC REF:448/2020). Participants under 18 therefore had the option of either obtaining or not obtaining parental consent given the sensitive nature of the survey topics including violence and sexual and reproductive health. Responses to disclosures of violence followed a standardized protocol and mandatory reporting guidelines in South Africa in accordance with the Children’s Act of 2005 [35]. All participants reporting experience of violence were offered a connection to a study counsellor. This was mandated for participants under 18 and optional for those over 18. In instances where abuse was confirmed in discussion with a counsellor, these cases were further referred to a Social Worker for ongoing care. Additionally, the research team monitored social harms throughout the study to identify and respond to any instances of abuse as a result of the study.

## Results

Table 1 presents the sample characteristics. A total of 534 participants were enrolled in the study, of which 60% were YPLWoH and 40% YPLWH. The mean sample age was 19 years with almost all (99.6%) identifying as Black African and as isiXhosa speakers (98.1%). Seven in ten participants identified as young women with a higher proportion among YPLWoH than YPLWH (78% vs. 58%, p < 0.001); 30% as young men, and two participants identified as “other” gender. A greater proportion of YPLWoH compared to YPLWH were living in informal dwellings (40% vs. 33%, p = 0.064), were worried about having enough food in the past month (61% vs. 37%, p < 0.001), and were not in education, employment or training (35% vs. 26%, p = 0.027). Half of the sample had an intimate partner at the start of lockdown (57% among YPLWoH vs. 39% among YPLWH, p < 0.001), but almost none lived with their partner (0.4%).

**Table 1:** Characteristics of young people enrolled in the BUDDY study, overall and by HIV status (n = 534)

Characteristic	Total sample (N = 534)	Youth living with HIV (N = 214)	Youth living without HIV (N = 320)	p-value
	n (%)	n (%)	n (%)	
***Sociodemographic***				
Current age, mean (SD)	19.0 (3.0)	19.0 (3.2)	19.1 (3.0)	0.74
Age categories				
13–14	63 (11.8%)	27 (12.6%)	36 (11.2%)	0.81
15–19	256 (47.9%)	104 (48.6%)	152 (47.5%)	
20–25	215 (40.3%)	83 (38.8%)	132 (41.2%)	
Sex				
Women	373 (69.9%)	124 (57.9%)	249 (77.8%)	< 0.001
Men	159 (29.8%)	90 (42.1%)	69 (21.6%)	
Black African race	532 (99.6%)	214 (100.0%)	318 (99.4%)	1.0
Primary language				
IsiXhosa	524 (98.1%)	207 (96.7%)	317 (99.1%)	0.071
English	7 (1.3%)	4 (1.9%)	3 (0.9%)	
Household type				
Formal dwelling	331 (62.1%)	143 (66.8%)	188 (58.9%)	0.064
Informal dwelling	198 (37.1%)	71 (33.2%)	127 (39.8%)	
Had a sexual partner during lockdown	261 (49.7%)	83 (39.0%)	178 (57.1%)	< 0.001
Lives with sexual partner	2 (0.4%)	1 (0.5%)	1 (0.3%)	1.00
NEET	168 (31.8%)	56 (26.3%)	112 (35.4%)	0.027
Worried about food, past month	274 (51.6%)	79 (37.1%)	195 (61.3%)	< 0.001
***COVID-related stressors***				
Self-isolated most or all the time	236 (44.6%)	114 (53.5%)	122 (38.6%)	< 0.001
Less support since lockdown	136 (26.0%)	62 (29.1%)	74 (23.8%)	0.17
Life lonelier because of COVID-19	186 (35.8%)	55 (25.9%)	131 (42.5%)	< 0.001
Moderately or extremely concerned about COVID illness	366 (69.6%)	127 (59.6%)	239 (76.4%)	< 0.001
Exposure to 3 or more stressors	182 (35.4%)	64 (30.2%)	118 (39.1%)	0.038
***Mental health***				
Clinically relevant depression (PHQ-9)	238 (45.2%)	102 (47.9%)	136 (43.5%)	0.32
Clinically relevant anxiety (GAD-2)	65 (12.4%)	29 (13.6%)	36 (11.5%)	0.47
Common mental disorders	247 (47.0%)	108 (50.7%)	139 (44.4%)	0.16

SD = standard deviation; p-values based on t-test, pearson’s chi-square test, and fisher’s exact test

NEET = not in education, employment, or training and includes participants ≥ 18 who are not in school and those < 18 who are not employed

2 participants (0.4%) selected “other” gender, 1 (0.2%) selected “coloured” race, 1 (0.2%) selected “white” race, 3 (0.6%) selected “Afrikaans” as primary language, and 4 (0.8%) selected “other” household type

Mental health measures indicate probable mental health disorders based on non-clinical screening. Scores on PHQ-9 range from 1 to 27 with scores ≥ 5 indicating at least mild depression. Scores ≥ 3 on GAD-2 indicate generalized anxiety. Common mental disorders refer to the presence of clinically relevant depression or anxiety

### Psychosocial wellbeing – COVID-related stressors and mental health

Across the four COVID-related stressors, YPLWoH reported higher levels than YPLWH of feeling that life was lonelier due to COVID (43% vs. 26%, p < 0.001), and being moderately or extremely concerned about COVID illness (76% vs. 60%, p < 0.001). YPLWH more often reported self-isolating (54% vs. 39%, p < 0.001) and having less contact with social support since lockdown (29% vs. 24%, p = 0.17). Finally, YPLWoH were more likely than peers living with HIV to have experienced three or more COVID stressors (39% vs. 30%, p = 0.038).

YPLWH and YPLWoH reported similar levels of depression and anxiety. Approximately 45% of the sample was indicated as having at least mild depression, and about one in six participants (12%) as having generalized anxiety (14% of YPLWH vs. 12% of YPLWoH). Combined, about half (47%) of participants were indicated as having a common mental disorder (51% of YPLWH vs. 44% of YPLWoH).

### Exposure to community, household, and intimate-partner violence

**Fig. 1:**
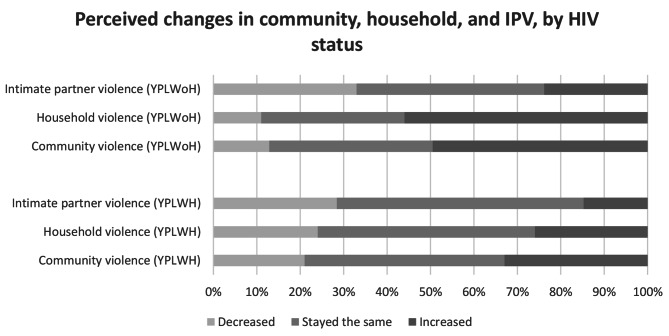
Perceived changes in community, household, and IPV, by HIV status YPLWoH = young people living without HIV; YPLWH = young people living with HIV

Community violence was perceived as having increased by almost half of YPLWoH (45%) compared to about a third among YPLWH (27%, p < 0.001; Table 2). Reported exposure to any household violence during the lockdowns was also significantly higher among YPLWoH as compared to YPLWH (36% vs. 23%, p = 0.006) and of those who experienced HV, it was perceived to have increased among a significantly greater proportion of YPLWoH (56% vs. 27%, p = 0.002). For those who reported having a partner during lockdown (n = 261), experience of any IPV was more frequently reported by YPLWoH than YPLWH (19% vs. 15%, p = 0.41) as was perceiving that IPV increased (15% among YPLWoH vs. 8% among YPLWH, p = 0.92), although results were not statistically significant. See Fig. 1 for a display of perceived changes in all forms of violence by participant HIV status.

**Table 2:** Prevalence of community, household, and intimate partner violence, overall and by HIV status

Variable	Total sample (N = 534), N (%)	YPLWH (N = 214), N (%)	YPLWoH (N = 320), N (%)	p-value
***Community violence (CV)***				
Perceived change in CV				
Decreased	76 (14.2%)	39 (18.2%)	37 (11.6%)	< 0.001
Stayed the same	193 (36.1%)	84 (39.3%)	109 (34.1%)	
Increased	204 (38.2%)	59 (27.6%)	145 (45.3%)	
***Household violence (HV)***				
Any HV	166 (31.1%)	50 (23.4%)	116 (36.2%)	0.006
Perceived change in any HV (N = 166)				
Decreased	25 (15.2%)	12 (24.5%)	13 (11.3%)	0.002
Stayed the same	62 (37.8%)	24 (49.0%)	38 (33.0%)	
Increased	77 (47.0%)	13 (26.5%)	64 (55.7%)	
***Intimate partner violence*** (N = 261)				
Any emotional IPV	30 (11.6%)	7 (8.5%)	23 (13.0%)	0.30
Any physical IPV	22 (8.5%)	5 (6.0%)	17 (9.6%)	0.33
Any sexual IPV	13 (5.0%)	2 (2.4%)	11 (6.2%)	0.19
Reproductive coercion	13 (5.0%)	4 (4.8%)	9 (5.1%)	0.93
Any IPV	45 (17.5%)	12 (14.6%)	33 (18.9%)	0.41
Perceived change in any IPV (N = 45)				
Decreased	9 (20%)	2 (16%)	7 (21%)	0.92
Stayed the same	13 (28%)	4 (33%)	9 (27%)	
Increased	6 (13%)	1 (8%)	5 (15%)	

52 participants (9.9%) responded ‘don’t know’ to perceived change in community violence and 9 (1.7%) were missing. Change in household violence was asked among those reporting witnessing any household violence, n = 166, with 2 responding ‘don’t know’. Intimate partner violence was asked of those participants reporting having a sexual partner during lockdown, n = 261, with 4 missing responses. Emotional violence includes restricted contact with family, insults or being made to feel bad, and threats to hurt you or someone you care about. Physical violence includes slapping, hitting, kicking, dragging, pushing, shoving, choking, or burning, as well as threats to use or actual use of a knife or gun. Sexual violence includes both physical force and pressure to have sexual intercourse. Reproductive coercion includes partner-related pressure or forced sex without a condom or birth control in order to get pregnant. Any IPV includes experience of emotional, physical, sexual violence or reproductive coercion. Change in IPV was asked among those reporting witnessing any IPV, n = 45, with 4 participants (12.5%) responding ‘don’t know’ and 13 responses missing

### Associations with community violence

In Table 3, we present the results of adjusted multinomial logistic regression for modelling perceived increased and maintained (i.e., stayed the same) community violence compared to decreased community violence (reference group). For the full study sample, none of the COVID-related stressors or mental health status were significantly associated with relative risk of increased or maintained levels of community violence. However, perceiving that CV increased was associated with several other risk factors among the full sample, including living in informal housing (RRR = 2.07; 95% CI = 1.12–3.83), being food insecure (RRR = 1.79; 95% CI = 1.00-3.20), and having an intimate partner during lockdown (RRR = 2.66; 95% CI = 1.46–4.87).

**Table 3:** Adjusted relative risk ratios (RRR) of community violence in relation to psychosocial wellbeing, overall and by HIV status

Variable	Total (N = 534)		YPLWH (N = 214)		YPLWoH (N = 320)	
	CV increased	CV stayed the same	CV increased	CV stayed the same	CV increased	CV stayed the same
	RRR (95% CI)	RRR (95% CI)	RRR (95% CI)	RRR (95% CI)	RRR (95% CI)	RRR (95% CI)
***COVID-related stressors***						
Self-isolated most/all the time	1.21 (0.69- 2.14)	1.17 (0.67-2.05)	1.34 (0.54-3.35)	1.40 (0.61- 3.21)	1.41 (0.62- 3.16)	1.75 (0.52- 2.67)
Less support since lockdown	1.50 (0.80-2.83)	0.86 (0.45-1.64)	1.66 (0.64-4.26)	0.74 (0.30-1.81)	1.53 (0.61- 3.16)	0.92 (0.34- 2.48)
Life lonelier because of COVID-19	1.12 (0.62- 2.02)	0.75 (0.45- 1.37)	0.87 (0.33-2.32)	0.72 (0.29-1.81)	1.10 (0.50- 2.43)	0.76 (0.33- 1.71)
Moderately/extremely concerned about COVID illness	0.73 (0.39-1.37)	0.83 (0.44- 1.54)	0.83 (0.34- 2.01)	0.81 (0.36-1.49)	0.45 (0.16- 2.43)	0.76 (0.26- 2.22)
***Mental health***						
Common mental disorders	0.97 (0.55- 1.72)	0.72 (0.41- 1.27)	0.63 (0.25- 1.59)	0.64 (0.27- 1.49)	1.46 (0.64 − 3.30)	0.79 (0.34- 1.83)
***Sociodemographic factors***						
Age	0.98 (0.87-1.09)	0.91 (0.81-1.02)	0.94 (0.79- 1.13)	0.84 (0.71-0.98)*	1.06 (0.89-1.26)	1.02 (0.85- 1.22)
Gender (ref: woman)	0.94 (0.52 − 1.70)	0.80 (0.45-1.44)	1.00 (0.39- 2.55)	0.92 (0.38-2.17)	1.10 (0.46- 2.63)	0.74 (0.29- 1.83)
NEET	1.71 (0.78- 3.66)	1.83 (0.85-3.96)	2.26 (0.70- 7.34)	2.64 (0.83- 8.37)	1.27 (0.43- 3.75)	1.25 (0.41- 3.78)
Household type (ref: formal dwelling)	2.07 (1.12–3.83) *	1.65 (0.90-3.03)	1.77 (0.64- 4.91)	1.91 (0.74- 4.93)	**2.05 (0.89-4.69) ±**	1.61 (0.69- 3.69)
Food insecurity	1.79 (1.00-3.20)*	1.34 (0.75-2.38)	1.46 (0.57-3.73)	0.91 (0.38- 2.21)	**1.84 (0.83- 4.07) ±**	1.75 (0.78 − 3.90)
Sexual partner during lockdown (ref: no partner)	2.66 (1.46–4.87)***	1.50 (0.83-2.74)	3.11 (1.17–8.19)*	1.29 (0.51- 3.24)	**2.06 (0.89- 4.76) ±**	1.54 (0.66- 3.59)

The multinomial regression compares perceived change in community violence (CV) since COVID-19 restrictions (increased, sustained) to decreased violence (referent). RRR = relative risk ratio; CI = confidence interval

Common mental disorders refer to the presence of clinically relevant depression or anxiety

NEET = ‘not in education, employment or training’

Significance is indicated as: ***<0.001, **<0.01, *<0.05, ± <0.1

When stratified by HIV status, living in informal housing (RRR = 2.05; 95% CI = 0.89-4.69), being food insecure (RRR = 1.84; 95% CI = 0.83-4.07) and having a partner during lockdown (RRR = 2.06; 95% CI = 0.89-4.76) remained associated with increases in community violence among YPLWoH. In contrast, among YPLWH, only having a partner during lockdown (RRR = 3.11; 95% CI = 1.17–8.19) was associated with increases in CV.

### Associations with household violence

Table 4 presents the results of the binomial regression for modelling any HV compared to none, and the multinomial regression for modelling increased and maintained (i.e., stayed the same) HV compared to decreased HV (reference outcome). Experiencing any household violence during lockdown was associated with having a common mental health disorder in the overall sample (aOR = 2.91; 95% CI = 1.93–4.39; Table 4), as well as when stratified by HIV status (aOR YPLWH = 3.39; 95% CI = 1.56–7.37; aOR YPLWoH = 3.04; 95% CI = 1.81–5.11). Being a man (aOR = 1.52; 95% CI = 1.04-3.00) and being food insecure was also associated with increased odds of experiencing any household violence within the overall sample (aOR = 1.72; 95% CI = 1.15–2.60), however the association between gender only remained for YPLWH (aOR = 2.04; 95% CI = 0.94-4.44) and the association with food insecurity only remained among YPLWoH (aOR = 1.67; 95% CI = 0.97-2.86). The only COVID stressor associated with decreased odds of experiencing household violence among the sample was being moderately or extremely concerned about COVID for YPLWH (aOR = 0.51; 95% CI = 0.25-1.02).

**Table 4:** Adjusted associations between household violence and psychosocial wellbeing, overall and by HIV status

Variable	Total (N = 534)			YPLWH (N = 214)			YPLWoH (N = 320)		
	Any HV aOR (95% CI)	HV increased RRR (95% CI)	HV stayed the same RRR (95% CI)	Any HV aOR (95% CI)	HV increased RRR (95% CI)	HV stayed the same RRR (95% CI)	Any HV aOR (95% CI)	HV increased RRR (95% CI)	HV stayed the same RRR (95% CI)
***COVID-related stressors***									
Self-isolated most/all the time	0.77 (0.51- 1.15)	1.93 (0.65-5.71)	2.52 (0.81-7.89)	0.83 (0.40- 1.74)	12. 49 (0.24-661.08)	36.86 (1.09-1251.34)*	0.87 (0.51-1.48)	2.73 (0.56-13.40)	2.93 (0.53-16.06)
Less support since lockdown	1.34 (0.85-2.11)	0.47 (0.16-1.35)	0.21 (0.07-0.65)**	1.19 (0.55- 2.61)	0.21 (0.01-5.97)	0.03 (0.00-0.81)*	1.47 (0.82- 2.62)	0.34 (0.08-1.54)	**0.22 (0.04-1.12)±**
Life lonelier because of COVID-19	1.16 (0.76-1.77)	1.65 (0.57-4.81)	1.35 (0.43-4.22)	0.87 (0.39- 1.97)	**22.81 (0.82-631.58)±**	1.88 (0.10-36.10)	1.14 (0.67- 2.32)	0.92 (0.21-3.97)	1.20 (0.25 − 5.90)
Moderately/extremely concerned about COVID illness	0.92 (0.59- 1.43)	2.26 (0.77-6.65)	1.73 (0.56-5.35)	**0.51 (0.25- 1.02)±**	0.22 (0.01-6.42)	0.14 (0.01-2.23)	1.25 (0.68- 2.32)	**3.48 (0.85-14.21) ±**	**5.09 (0.99-26.12)±**
***Mental health***									
Common mental disorders	2.91 (1.93–4.39)***	1.07 (0.36-3.15)	0.59 (0.19-1.81)	3.39 (1.56–7.37)**	7.70 (0.35-168.11)	0.91 (0.09-9.46)	3.04 (1.81–5.11)***	0.82 (0.18-3.77)	0.41 (0.08-2.07)
***Sociodemographic factors***									
Age	0.99 (0.90- 1.08)	1.12 (0.88-1.42)	1.12 (0.87-1.44)	0.96 (0.83-1.15)	1.29 (0.63-2.64)	1.64 (0.92-2.94)	1.01 (0.90- 1.14)	0.95 (0.69-1.31)	0.93 (0.66-1.32)
Gender (ref: woman)	**1.52 (0.98-2.36)±**	1.20 (0.46-3.19)	0.65 (0.22-1.91)	**2.04 (0.94- 4.44) ±**	58.30 (1.29-2622.88)*	**24.29 (0.68-872.92) ±**	1.61 (0.91 − 2.90)	0.99 (0.25-3.96)	0.49 (0.10-2.41)
NEET	1.76 (1.04- 3.00)*	2.50 (0.58-10.74)	6.01 (1.31–27.81)*	2.74 (1.05–7.17)**	**181.81 (1.31-25256.97)±**	**61.73 (0.53-7255.57) ±**	1.38 (0.71- 2.69)	3.21 (0.48-21.53)	10.23 (1.36–78.99)*
Household type (ref: formal dwelling)	1.09 (0.73- 1.63)	0.70 (0.27-1.84)	0.52 (0.18-1.45)	1.66 (0.79- 3.51)	**0.03 (0.00-0.68)±**	0.25 (0.03-2.84)	0.90 (0.54- 1.48)	1.27 (0.34-4.76)	0.57 (0.13-2.46)
Food insecurity	1.72 (1.15–2.60)*	1.37 (0.49-3.78)	2.09 (0.70-6.27)	1.29 (0.62- 2.68)	0.06 (0.00-1.75)	0.44 (0.04-4.81)	**1.67 (0.97- 2.86) ±**	1.93 (0.44-8.46)	3.13 (0.61-15.97)
Sexual partner during lockdown (ref: no partner)	0.95 (0.61- 1.47)	0.63 (0.20-1.92)	0.28 (0.09-0.94)*	0.68 (0.31- 1.54)	**0.03 (0.00-2.01)±**	0.01 (0.00-0.57)*	0.94 (0.54- 1.63)	1.28 (0.31-5.26)	0.70 (0.15-3.32)

Binomial logistic regression compares any household violence experience to no household violence experience (referent). The multinomial regression compares perceived change in household violence since COVID-19 restrictions (increased, stayed the same) to decreased violence (referent). OR = odds ratio; RRR = relative risk ratio; CI = confidence interval

Common mental disorders refer to the presence of clinically relevant depression or anxiety

NEET = ‘not in education, employment or training’

Significance is indicated as: ***<0.001, **<0.01, *<0.05, ± <0.1

Among those who reported witnessing HV, NEET status was positively associated with perceiving that household violence levels stayed the same compared to a decrease among YPLWH (RRR = 24.29; 95% CI = 0.53-7255.57) and YPLWoH (RRR = 10.23; 95% CI = 1.36–78.99). Having a sexual partner was protective among the full sample (RRR = 0.28; 95% CI = 0.09-0.94) and YPLWH (RRR = 0.01; 95% CI = 0.00-0.57) in terms of perceiving that household violence levels stayed the same compared to decreased. Among the COVID stressors, having less contact with social support since lockdown (RRR = 0.21; 95% CI = 0.07-0.65) was associated with reduced relative risk of perceiving household violence stayed the same among the full sample, as well as among YPLWH and YPLWoH. Finally, being moderately or extremely worried about COVID illness was associated with levels of household violence remaining the same among YPLWoH (RRR = 5.09; 95% CI = 0.99-26.12) and increasing (RRR = 3.48; 95% CI = 0.85-14.21) but not among peers living with HIV.

## Discussion

While numerous articles and reviews have called attention to the ways in which COVID-19 may have contributed to increased GBV [20, 36–38], few studies thus far have empirically examined the role of the theorized psychosocial impacts of the pandemic – such as social isolation and increased stress on experience of violence. Our study adds to evidence about the potential effects of COVID-19 on young people’s experience of violence by examining these associations and contributing new nuances to our understanding through its investigation of differences by HIV status, and evaluation of violence at multiple levels of society – community, households, and intimate partnerships. We observed concerning levels of perceived increases in community and household violence since the March 2020 COVID lockdown in South Africa, but less exposure to IPV among young people, who rarely lived with their partners during lockdown. While we also saw limited evidence that COVID-specific stressors were strongly associated with changes in violence, the finding that socioeconomic status and mental health appeared to be more important to risk, is an important contribution for considerations of future responses in a setting like South Africa.

In contrast to previous evidence linking living with HIV with increased vulnerability to violence [10, 39, 40], we found that perceived increases in violence were more common among those living without HIV, as well as higher levels of socioeconomic vulnerabilities such as living in informal housing, and food insecurity among this population. While we do not know the specific underlying reasons for these associations, it may be related to enhanced government social support and/or comparatively greater contact with health care providers during lockdowns among our sample of YPLWH who were in care at the time of recruitment. The government of South Africa has a commitment to ensuring that people living with, or at risk of, HIV have access to cash transfers. Although studies indicate mixed impacts of disability grants on the well-being of people living with HIV, they are eligible to receive a cash transfer if they qualify as being disabled due to the disease [41, 42]. They are also offered free care, both of which had fairly high coverage in 2018, e.g. 17.5 million receiving any form of government cash transfer, and 68% of those diagnosed with HIV are on treatment [43]. While our analyses of the broader BUDDY study findings examining access to healthcare during COVID found greater unmet need for sexual and reproductive health services among YPLWH [24], a systematic review of the impact of COVID on HIV services in South Africa indicated limited evidence of disruptions in ART provision in the country [44]. In the current study, YPLWH were also less likely to report that life was lonelier due to COVID and less concerned about COVID illness, which could also be related to greater contact with the health care system.

We further found that indicators of socioeconomic status – i.e., living in informal housing, being food insecure, and being out of school or unemployed, i.e., ‘NEET’ – were more often associated with perceived increases in violence than COVID-related stressors. This points towards the fact that high levels of pre-existing vulnerabilities to violence in the country may have outweighed the added psychosocial stress of COVID. This is in spite of programs such as the Social Relief of Distress grant, which was introduced at the start of the COVID-19 pandemic, to provide food parcels or vouchers for food to those in need [45]. Similarly, findings in a systematic review of factors associated with IPV during COVID identified unemployment, low socioeconomic status, and overcrowding as associated risk factors [1], and have been qualitatively confirmed at the household level through research in the South African setting with women and children [46]. Despite being defined as an upper middle-income country by the World Bank, South Africa has higher than average levels of poverty than other countries in this category [47]; is ranked as having the highest gini coefficient in the world (i.e. global measure of inequality) [48]; and South Africans face incredibly high rates of unemployment with youth at greater risk (42.1% youth vs. 20.2% adults are unemployed) [49]. In addition, prior to COVID, studies began identifying an association between food insecurity, in particular, with both experience and perpetration of GBV [15, 50–52], confirming our findings. The relationship between the built environment, inclusive of housing, and violence has also been explored, although primarily qualitatively [53–56]. One such study described how penetrable housing material, lack of resources such as water and toilet facilities which force people out of the home, and housing size, which reduces privacy and forces individuals outside, are key reasons for increased violence exposure among informal housing residents [56]. Future research should aim to unpack whether factors such as overcrowding, permeability, or other pathways explain the identified relationship.

Like all research, this study is not without limitations. For one, the cross-sectional nature of the study inhibits our ability to determine the direction of associations. For example, those with existing mental ill-health may be at increased risk of violence, or mental-ill health may be a consequence of exposure to violence. Recall bias given the delay in data collection from time of lockdown is also a concern, however given the severity of lockdown measures in South Africa and interviewers emphasis on the date when lockdown began, it is unlikely that individuals were unable to recall this period. While conducting telephonic interviews was sometimes necessary due to COVID, our use of both telephonic and in-person surveys may have contributed to differential response bias. This may have been a particular concern in terms of sharing sensitive information over the phone in cases where participants did not have full privacy. A synthesis of challenges and opportunities related to remote data collection methods used frequently during COVID, however, identified interviewer-led telephonic interviews as being less prone to measurement error than other remote survey methods (e.g. SMS or other automated surveys) and suggested that in some cases participants may be more open over the phone [57]. To enhance safety and reduce reporting bias, participants surveyed over the phone were requested to identify a private space for the interview and requested to confirm this upon participation. Prior to asking about experiences of violence, interviewers also introduced the upcoming topic and reminded participants of mandatory reporting laws, as well as their access to counselling services. Finally, the small sample size, which was not powered to detect changes in violence, may have limited our ability examine associations with COVID-related stressors, in particular with experience of IPV given its lower reported levels. Notwithstanding these limitations, to the best of our knowledge the current study is one of the first designed to explore differences between YPLWH and YPLWoH adding a valuable contribution to our understanding of how various forms of violence differed among these populations and their associated risk and protective factors.

## Conclusion

Our study is the first that we know of to look at more comprehensive range of forms of GBV among young men and women living with and without HIV and to examine associations with theorized COVID-specific risk factors. Findings from this peri-urban Cape Town context indicates that socioeconomic status and mental health may have played a larger role than COVID-related stress on impacting changes in community and household violence from the perspective of young people. The potential role of these factors was particularly apparent for young people living without HIV, who reported being more exposed to increased violence during the pandemic in contrast to peers living without HIV. Our findings confirm a need for improving basic housing, food security, and mental health as key care to mitigate violence exposures overall, as well as during community-wide public health crises such as those related to future pandemics or climate change. In addition, longitudinal and mixed-methods research studies are needed to examine and unpack the specific mechanisms responsible for increases in GBV and other forms of violence among young people in order to better inform policies for prevention and response.

### Electronic supplementary material

Below is the link to the electronic supplementary material.

**Additional file 1**. Prevalence of community, household, and intimate partner violence, overall and by gender.

## Data Availability

The datasets used and/or analyzed during the current study are available from the corresponding author on reasonable request.

## List of abbreviations

CV: Community violence
HV: Household violence
IPV: Intimate–partner violence
GBV: Gender–based violence
NEET: Not in education, employment, or training
YPLWH: Young people living with HIV
YPLWoH: Young people living without HIV
