# Supplementary material for: Associations between psychosocial wellbeing and experience of gender-based violence at community, household, and intimate-partner levels among a cross-sectional cohort of young people living with and without HIV during COVID-19 in Cape Town, South Africa
Source: BMC Public Health. 2023 Oct 27;23:2115. doi: 10.1186/s12889-023-16945-5 (PMC10612288; doi:10.1186/s12889-023-16945-5)
Supplement: Supplementary file 1 — Additional file 1. Prevalence of community, household, and intimate partner violence, overall and by gender. [file 12889_2023_16945_MOESM1_ESM.docx]

**Additional file 1. Prevalence of community, household, and intimate partner violence, overall and by gender**

| **Variable** | | **Total sample, N (%)** | **Female, N (%)** | **Male, N (%)** | **Other, N (%)** | **p-value** |
| --- | --- | --- | --- | --- | --- | --- |
|  | ***Community violence (CV)*** | | | | | |
|  | Perceived change in CV | | | | | |
| Decreased | | 76 (16.1%) | 49 (14.8%) | 27 (19.4%) | 0 (0.0%) | 0.37 |
| Stayed the same | | 193 (40.8%) | 137 (41.3%) | 56 (40.3%) | 0 (0.0%) |  |
| Increased | | 204 (43.1%) | 146 (44.0%) | 56 (40.3%) | 2 (100.0%) |  |
|  | ***Household violence (HV)*** | | | | | |
| Any HV | | 166 (32.9%) | 115 (32.5%) | 49 (32.9%) | 2 (100.0%) | 0.13 |
|  | Perceived change in any HV (N=165) | | | | | |
| Decreased | | 25 (15.2%) | 16 (14.2%) | 9 (18.4%) | 0 (0.0%) | 0.50 |
| Stayed the same | | 62 (37.8%) | 46 (40.7%) | 16 (32.7%) | 0 (0.0%) |  |
| Increased | | 77 (47.0%) | 51 (45.1%) | 24 (49.0%) | 2 (100.0%) |  |
|  | ***Intimate partner violence*** (N=261) | | | | | |
| Any emotional IPV | | 30 (11.6%) | 21 (11.5%) | 8 (10.5%) | 1 (100.0%) | 0.021 |
| Any physical IPV | | 22 (8.5%) | 16 (8.8%) | 5 (6.5%) | 1 (100.0%) | 0.004 |
| Any sexual IPV | | 13 (5.0%) | 9 (4.9%) | 4 (5.2%) | 0 (0.0%) | 0.97 |
| Reproductive coercion | | 13 (5.0%) | 8 (4.4%) | 5 (6.6%) | 0 (0.0%) | 0.74 |
| Any IPV | | 45 (17.5%) | 29 (16.1%) | 15 (19.7%) | 1 (100.0%) | 0.074 |
|  | Perceived change in any IPV (N=45) | | | | | |
| Decreased | | 9 (28%) | 8 (36%) | 1 (10%) | - | 0.37 |
| Stayed the same | | 13 (41%) | 7 (32%) | 6 (60%) | - |  |
| Increased | | 6 (19%) | 4 (18%) | 2 (20%) | - |  |

52 participants (9.9%) responded ‘don’t know’ to perceived change in community violence and 9 (1.7%) were missing. Change in household violence was asked among those reporting witnessing any household violence, n=166, with 2 responding ‘don’t know’. Intimate partner violence was asked of those participants reporting having a sexual partner during lockdown, n=261, with 4 missing responses. Emotional violence includes restricted contact with family, insults or being made to feel bad, and threats to hurt you or someone you care about. Physical violence includes slapping, hitting, kicking, dragging, pushing, shoving, choking, or burning, as well as threats to use or actual use of a knife or gun. Sexual violence includes both physical force and pressure to have sexual intercourse. Reproductive coercion includes partner-related pressure or forced sex without a condom or birth control in order to get pregnant. Any IPV includes experience of emotional, physical, sexual violence or reproductive coercion. Change in IPV was asked among those reporting witnessing any IPV, n=45, with 4 participants (12.5%) responding ‘don’t know’ and 13 responses missing.
